# Production and bioprocessing of Taxol from *Aspergillus niger*, an endophyte of *Encephalartos whitelockii,* with a plausible biosynthetic stability: antiproliferative activity and cell cycle analysis

**DOI:** 10.1186/s12934-024-02356-7

**Published:** 2024-03-12

**Authors:** Asmaa Gamal, Eman Fikry, Nora Tawfeek, Azza M. El-Shafae, Ashraf S. A. El-Sayed, Maher M. El-Domiaty

**Affiliations:** 1 Pharmacognosy Department, Faculty of Pharmacy, Zagazig University, Zagazig, 44519 Egypt; 2 Enzymology and Fungal Biotechnology Lab, Botany and Microbiology Department, Faculty of Science, Zagazig University, Zagazig, 44519 Egypt

**Keywords:** Taxol, *Aspergillus niger*, *Encephalartos whitelockii*, Antiproliferative activity, Wound healing, Cell cycle analysis

## Abstract

The biosynthetic potency of Taxol by fungi raises their prospective to be a platform for commercial production of Taxol, nevertheless, the attenuation of its productivity with the fungal storage, is the challenge. Thus, screening for a novel fungal isolate inhabiting ethnopharmacological plants, with a plausible metabolic stability for Taxol production could be one of the most affordable approaches. *Aspergillus niger* OR414905.1, an endophyte of *Encephalartos whitelockii*, had the highest Taxol productivity (173.9 μg/L). The chemical identity of the purified Taxol was confirmed by HPLC, FTIR, and LC–MS/MS analyses, exhibiting the same molecular mass (854.5 m/z) and molecular fragmentation pattern of the authentic Taxol. The purified Taxol exhibited a potent antiproliferative activity against HepG-2, MCF-7 and Caco-2, with IC_50_ values 0.011, 0.016, and 0.067 μM, respectively, in addition to a significant activity against *A. flavus*, as a model of human fungal pathogen. The purified Taxol displayed a significant effect against the cellular migration of HepG-2 and MCF-7 cells, by ~ 52–59% after 72 h, compared to the control, confirming its interference with the cellular matrix formation. Furthermore, the purified Taxol exhibited a significant ability to prompt apoptosis in MCF-7 cells, by about 11-fold compared to control cells, suppressing their division at G2/M phase. Taxol productivity by *A. niger* has been optimized by the response surface methodology with Plackett–Burman Design and Central Composite Design, resulting in a remarkable ~ 1.6-fold increase (279.8 μg/L), over the control. The biological half-life time of Taxol productivity by *A. niger* was ~ 6 months of preservation at 4 ℃, however, the Taxol yield by *A. niger* was partially restored in response to ethyl acetate extracts of *E. whitelockii*, ensuring the presence of plant-derived signals that triggers the cryptic Taxol encoding genes.

## Introduction

Cancer diseases are currently ranked as the second notable reason of mortality after cardiovascular disease [1, 2]. Chemotherapy has been recognized as one of the most profound treatment protocol for cancer, and Taxol is one of the most prescribed drugs. Taxol, a diterpenoid that was initially isolated from *Taxus brevifolia* with a significant anticancer activity by arresting the tumor cell cycle at the G2/M phase [3, 4]. Taxol had a powerful anticancer activity against a wide-range of tumor cell lines including breast, ovarian, hepatic, leukemia, lung tumors, and polycystic kidney disease [5, 6]. The influential activity of Taxol against various tumor cells is mainly due the unique affinity to bind with N-terminal region of β-tubulin, stopping the depolymerization of the microtubules, so, blocking the mitotic division, with an ultimate arrest to the cell cycle of tumor cells [7, 8]. Taxol was originally derived from *T. brevifolia* bark*,* nonetheless, its significantly low yield (~ 0.001%), unpredicted fluctuations and reproducibility, due to the natural environmental changes that could have a negative effect on the Taxol biosynthetic machinery by *T. brevifolia,* are the challenge for this approach [9, 10, 11]. Semisynthetic technology based on *Taxus baccata* for production of the intermediate 10-decaetylbaccatin III, followed by tailoring enzymes to synthesize Taxol, has been used as an authenticated approach for the commercial Taxol production [12], however, the heterogeneity of these intermediates are the major obstacle. Interestingly, the fungal metabolic potency of Taxol production raise the hope for implementation of this approach commercially, for the feasibility of manipulating their yield, independence on the environmental conditions, rapid growth, and cost-effectiveness [13, 14]. Numerous endophytic fungi were reported to possess the Taxol producing metabolic potency [15–20]. The biosynthetic pathway of Taxol in fungi started with cyclization of geranylgeranyl diphosphate to taxa-4(5),11(12)-diene by the action of taxadiene synthase, then hydroxylation of taxadiene nucleus by the cytochrome P450-monooxygenases, as reviewed in details by our previous studies [21]. However, the anticipation of fungi to be a commercial approach for Taxol production has been confronted by the attenuation of Taxol productivity by fungi with the storage and multiple subculturing [16, 21–24]. The machinery of Taxol biosynthesis in fungi is usually encoded by gene cluster located on different domains on the fungal genome, and the expression of these cluster become cryptic under standard lab conditions, due to the lack or dilution of the transcriptional signals to synchronize the expression of these genes [18, 24–28]. Thus, screening for a metabolically stable Taxol producing fungal isolate from different medicinal plants is the objective.

Medicinal plants of ethno-pharmaceutical uses could be a repository for novel endophytic fungi with unique metabolic stability and sustainability for bioactive secondary metabolites. The genus *Encephalartos* has been recognized as one of the well-known pharmaceutically valuable plants, the second-largest genus of the family Zamiaceae, predominantly developed in south of the Sahara Desert [29–31], tropical Africa, over 50% of the genus has been present in South Africa [32]. *Encephalartos* has a significant ethno-pharmacological value, exhibiting cytotoxic and antifungal properties [33–35], however, the identity of the endophytic fungi from this genus remains ambiguous. Thus, the objective was to search for a potential endophytic fungal isolate from *Encephalartos whitelockii*, with a reliable biosynthetic stability for Taxol production, and to maximize the yield of Taxol via Surface Response Methodology optimization bioprocessing.

## Material and methods

### Plant sample collection, and isolation of the fungal endophytes

Fresh leaves samples of *Encephalartos whitelockii* were obtained in March 2022 from El-Abd Garden in Giza, Egypt, for isolation of their endogenous fungal endophytes. The plant was kindly identified by Dr. Therese Labib, a Plant Taxonomist at Orman Botanical Garden in Giza, Egypt, and the sample voucher was deposited at the Department of Pharmacognosy Herbarium, Faculty of Pharmacy, Zagazig University, with ID# ZU-Ph-Cog-608 was deposited. The leaves were gathered and sterilized by 70% ethanol for 2 min, then by 2.5% sodium hypochlorite for 2 min, rinsed twice with distilled water. The leaves were carefully segmented into 1 cm × 1 cm, and placed onto the surface of potato dextrose agar (PDA) [20] (200 *g*/L potato extract, 20 *g*/L glucose, and 20 *g*/L agar in distilled water, with 0.1 *g* of chloramphenicol). The plates were incubated at 30 ℃ for 10 days [16, 36], and the emerged colonies of fungi were purified on the same media, and the developed fungi were identified according to their morphological features [37–39]

### Screening for Taxol production by the endophytic fungi

The endophytic fungi from the leaves of *E. whitelockii* were grown on potato dextrose broth (PDB) [16, 20]. A plug of 6-day-old culture on PDA of each isolate was inoculated into PDB medium, the cultures were incubated at 30 ℃ for 10 days, filtered, the filtrates were centrifuged at 5000 rpm, followed by extraction with ethylacetate [40, 41]. The collected organic phase of solvent was dried by rotary evaporation, the resulting residues were dissolved in methanol. The Taxol productivity was evaluated by Merck 1 mm (20 × 20 cm) pre-coated silica gel TLC plates (Silica gel 60 F254, KGaA, Darm, Germ.) [20, 42], with methylene chloride/ methanol/dimethyl formamide of 90:9:1 (v/v/v). The TLC plates were UV-illumined at λ_254 _nm, to visualize the putative Taxol spots, as well as the plates were sprayed with a 1% vanillin sulfuric acid solution, and gently heated for 24 h. The putative Taxol sample gave the same bluish color and mobility rate of the authentic one (Cat. # T7402), were considered. The putative silica spots containing Taxol were scraped off for Taxol extraction [16, 20, 21]. The concentration and purity the extracted Taxol were evaluated using HPLC (Agilent Technology, G1315D) of Eclipse Plus RP-C18 column (Cat. # 959963-902) with methanol/ acetonitrile/water (25:35:40, v/v/v) at 1.0 mL/min for 20 min. The fractions of Taxol were scanned using a photodiode array detector (DAD) from 200 to 500 nm, and the concentrations of the putative Taxol were determined, compared to the authentic one at λ_227_ nm [20, 43].

### Spectroscopic analyses of Taxol

The absorbance of the putative Taxol from the most potent fungal isolate was measured by UV–Vis spectrophotometry at wavelength range 200–400 nm. The concentration and purity of the extracted sample were assessed, compared to the authentic Taxol, with methanol for zeroing the spectrophotometer [16]. The Infra-Red (FT-IR) spectra of the sample were determined using a JASCO, FTIR 6100 Spectrophotometer, sample was pulverized in KBr pellets, and the spectra were recorded from 4000 to 500 cm^−1^.

The identity of the purified Taxol was resolved by the LC–MS/MS with a Thermo Scientific LCQ Deca mass spectrometer and Hypersil Gold aQ (C18 column) equipped with a positive ion mode electrospray source. The gradient elution mobile phase system of solution A (0.1% formic acid) and B (acetonitrile in 0.1% formic acid), was used with at 0.2 mL/min for 40 min, with a mobile phase B gradient ranging from 2 to 98% [16, 17, 21]. The chemical features of the committed signals were assessed by analyzing their fragmentation pattern with the NIST mass spectral library.

### Molecular identification of the potent Taxol-producing fungal isolate

The potent Taxol-producing fungal isolate was molecularly identified based on the internal transcribed spacers (ITS) sequence [44, 45]. The fungal genomic DNA (gDNA) was extracted by CTAB reagent [46], used as a PCR template with the primers ITS5 5ʹ-TCCTCCGCTTATTGATATGC-3ʹ and ITS4 5ʹ-GAAGTAAAAGTCG TAACAAGG-3ʹ [47]. The reaction of PCR contains 10 µL of 2X TOPsimple^™^ DyeMIX-nTaq (Cat. # P510T), 1 µL gDNA, 1 µ of each primer (5 pmol) in 20 µL total volume. The PCR program was denaturation at 95 ℃ for 2 min, followed by 35 cycles of denaturation at 95 ℃ for 30 s, annealing at 55 ℃ for 40 s, and extension at 72 ℃ for 40 s, and then 72 ℃ for 2 min in final extension. The PCR amplicons were analyzed by 1.5% agarose gel, and the amplicons were purified, sequenced, and the obtained sequences were non-redundant BLAST searched, then imported into MEGA X software, and aligned by the ClustalW algorithm. Finally, the phylogenetic analysis was assessed using the neighbor-joining with 100 bootstrap replicates [48].

### Antiproliferative activity of the crude ethylacetate and purified Taxol extracts

The anticancer activity of the crude ethyl acetate extract, and purified Taxol of the potent fungal isolate towards the human hepatocellular (Hep-G2), breast (MCF-7) and intestinal (Caco-2) carcinoma, compare to the Vero cells was assessed by MTT assay [49]. The 96-well microtiter plate was initially inoculated with 2 × 10^3^ cells/ well, incubated at 12 h at 37 ℃, then ethyl acetate and purified Taxol were added to at different concentrations, then further incubated of the plate for 48 h at 37 ℃ at 4% CO_2_. The MTT reagent was added to the plate, incubated for 6 h, the resulting purple formazan compound was assayed at λ_570 _nm. The IC_50_ values were represented by the concentration of Taxol inhibits the cell growth by 50%, compared to the control group (without drug). For the normal cell line (Vero), the CC_50_ value was determined by the drug concentration reducing the initial cellular growth by 50%. The selectivity index (S.I.) was expressed by the value of CC_50_ of the Vero cell to the IC_50_ value towards the cancer cells.

### Biological activity-guided assay of ethylacetate extract and putative Taxol extracts

The antifungal activity of ethyl acetate extract of the potent fungi and the putative purified Taxol sample was evaluated against *Aspergillus flavus*, as a model human fungal pathogen [50, 51]. The fungal spores was mixed with PDA prior plate pouring, incubated for 6 h, then different concentrations (0.1, 0.5, 2.5, 5, and 10 µg/mL) of the extracted Taxol were injected to the wells of the plate cultures, incubated at 28 ℃ for 5 days, and the diameter of the fungal inhibition zones was determined. The negative control was 1% DMSO. Triplicates of the experiments were performed, and the results were presented by the mean ± standard deviation. Minimum inhibitory concentration (MIC) was assessed as the lowest extract concentration effectively inhibits the visual fungal growth, based on the inhibition zone diameters [52].

### Anti-wound healing activity of the extracted Taxol

The effect of purified Taxol from the potent fungus on the stopping the cellular migration of MCF-7 and HepG-2 tumor cells was determined. The 12-wells microtiter plate were seeded with at 4 × 10^6^ cells to, allowed to grow to a confluent monolayer, then a wound was made on the cell layer, the plate was rinsed with PBS, and amended with fresh medium with the extracted Taxol at IC_25_ value, compared to 2% DMSO as a negative control. The culture was incubated at 37 ℃ at 4% CO_2_, and the wound closure were monitored and imaged by phase-contrast microscopy. The healing efficiency of wound was calculated the percentage of a scratch area in drug-treated cells, compared to untreated cells.

### Apoptosis and cell cycle analyses of the MCF-7 cells due to the putative Taxol

The apoptotic analysis of MCF-7 cells was assessed by Annexin- Apoptosis Detection Kit (Catalog #: K101-25). This assay based on the reaction of Annexin V protein with the externalized phosphatidylserine (PS) of the cells, with the initial stages of apoptosis, and the complex of Annexin V-PS can be easily measured by flow cytometry [53]. The MCF-7 cells were seeded to a 96-well plate culture at 2 × 10^7^ cells/well, treated with the purified Taxol, incubated for 24 h. The cultured cells were harvested, washed with 1 mL of PBS, and 200 μL of 1X annexin-binding buffer was added. After the addition of Annexin V-FITC and PI to the cells, the mixture was incubated in total darkness for 15 min. The Annexin-PS complex was detected (Ex, λ_488 _nm; Em, λ_530 _nm) using a FITC signal detector.

The analysis of MCF-7 cell cycle in response to purified Taxol was assessed by propidium Iodide (PI) assay Kit (Cat #. ab139418). Briefly, the 48-wells microtiter plate were seeded with the cells, incubated for 12 h at 37 ℃, Taxol was added at IC_25_ value, and then the cultures were incubated for 48 h. The cells were harvested by centrifugation for 5 min at 2000 rpm, fixed in ice-cold 70% ethanol for 2 h at 4 ℃, and then the cells were rehydrated with 1 mL of PBS, and stained with PI solution containing 5 μg/mL RNase, for 30 min in dark. Subsequently, the cellular DNA were analyzed using flow cytometry, with excitation at λ_493_ nm and emission at λ_636_ nm, using Flow Jow software package for calculating the of G0-G1, S, and G2-M cells percentages.

### PCR mining of baccatin III-3-O-(3-Amino-3-phenylpropanoyl) transferase

The Taxol molecular biosynthetic blueprint was assessed by PCR mining of baccatin III-3-O-(3-amino-3-phenylpropanoyl) transferase (*bapt)*, as a rate-limiting gene of Taxol synthesis. The extracted fungal DNA was used as a PCR template for *bapt* amplification with the primers 5ʹ-TGAGGACCTCCATCTCTTCAT-3ʹ; 5ʹ-TACACATTCGCTCCC ACAAC-3ʹ. The PCR mixture contains 10 μL of TOPsimple^™^ DyeMIX-nTaq (Cat.# P510T), 2 μL DNA, and 1 μL of each primers (10 pmol) in 20 μL total volume, using the Thermal Cycler 006 (A&E Lab Co. Ltd. England). The PCR was programed to denaturation at 94 ℃ for 2 min, followed by denaturation at 94 ℃ for 20 s, annealing at 51 ℃ for 30 s, and extension at 72 ℃ for 30 s, for 35 cycles, and 72 ℃ for 2 min as a final extension. The PCR amplicons were checked by 1.5% agarose gel. Subsequently, these amplicons were purified and sequenced with the same primers. The obtained sequence of *bapt* was non-redundant BLAST searched and aligned using Clustal W [54], phylogenetic analysis was conducted by neighbour-joining [55].

### Nutritional bioprocessing of *A. niger* for optimizing the Taxol yield by plackett–burman and central composite designs

Various nutritional requirements including maltose, sucrose, lactose, peptone, soytone, yeast extract, ammonium tartrate, sodium acetate, sodium nitrate, calcium chloride, magnesium sulfate, potassium hydrogen phosphate, ammonium sulfate, fluconazole, methyl jasmonate, cysteine, phenylalanine, methionine and glycine, were optimized by Plackett–Burman design for maximizing the Taxol yield of *A. niger*. The nineteen parameters were symbolized by (+ 1) and (− 1), as high and low levels, respectively. Response Surface Methodology has been recognized as an effective approach for evaluating the interactions of the independent parameters and their consequences on productivity of Taxol by fungi [40]. The first-ordered polynomial model was calculated from the determination coefficient (R2), and F-test according to the following equation:

$$Y = \beta 0 + \Sigma \beta iXi$$

Y is predicted yield of Taxol, Xi an independent variable, *β*i the linear coefficient, and *β*0 the model intercept. Three replicates were performed for each trial. The most influential variables derived from the Plackett–Burman Design (PBD), influencing on the Taxol production by *A. niger* were optimized by the Central Composite Design (CCD) [44, 83].

The second-ordered polynomial model has been utilized for predicting the optimum storage conditions for Taxol production according to the following equation:

$$Y \, = \beta_{0} + \sum \beta_{i} {\text{\rm X}}_{i} + \sum \beta_{ii} x_{ii} + \sum \beta_{ij} {\text{\rm X}}_{ij} ,$$

β_i_ the variables regression coefficient, β_ii_ the regression coefficient of square effects, β_ij_ the regression coefficient of interactions.

### Taxol productivity by *A. niger* with the storage, potency of restoring their productivity by the plant extracts

The metabolic biosynthetic stability of Taxol by *A. niger*, with the storage was assessed. The original fungal culture was preserved as slope cultures at 4 ℃ for 10 months, and the Taxol productivity by the fungus was monthly assessed by growing at the standard conditions, then Taxol was extracted and quantified by the TLC and HPLC [14, 21].

The impact of different extracts of *E. whitelockii* namely; dichloromethane, ethyl acetate, and ethanol on induction of the Taxol productivity by *A. niger* was assessed. The fresh leaves (5 *g*) of *E. whitelockii* were minced in 50 mL of each solvent, and the mixture was stored at 4 ℃ for 12 h, and the resulting extracts were filtered, centrifuged at 5000 rpm for 10 min, and the extracts were concentrated to 10 mL. Different concentrations of the prepared extracts were added to the 5-days old pre-cultures of *A. niger,* and further incubated for 14 days, and then Taxol was extracted and quantified [36]. Blank media without *A. niger,* with the plant extracts, and control cultures of *A. niger* without plant extracts, were used.

### Fungal deposition

The internal transcribed sequence of *A. niger*, an endophyte of *E. whitelockii*, was deposited to Genbank with accession number OR414905.1.

### Statistical analysis

Triplicates of each experiment were executed, and the results were represented by the mean ± standard deviation. The significance and F-test statistical analysis was determined the one-way ANOVA by using Prism Version 6.0 (GraphPad Software, Inc., CA, USA).

## Results

### Isolation of the endophytes of *Encephalartos whitelockii,* screening for Taxol production

**Fig. 1:**
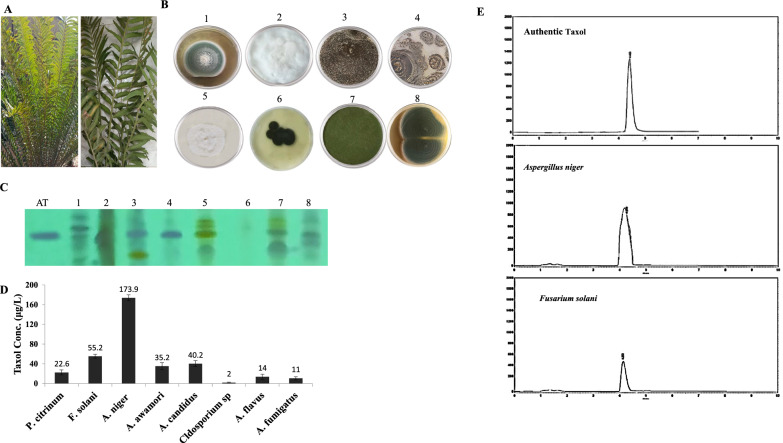
Isolation and screening for the endophytic fungal isolates of *E. whitelockii* leaves. **A** Morphological view of *E. whitelockii*. **B** Plate cultures of the recovered fungal endophytes. (1. *Penicillium citrinum, 2. Fusarium solani, 3. Aspergillus niger,* 4. *A. awamori*, 5. *A. candidus*, 6. *Cladosporium* sp., 7.*A. flavus* and 8. *A.fumigaus*). **C** The TLC plate of the extracted Taxol from the fungal isolates normalizing to authentic Taxol. **D** Yield of Taxol as quantified from the TLC chromatograms.** E** HPLC chromatogram of the TLC purified Taxol from *A. niger,* and *F. solani*, compared to the authentic Taxol at retention time 4.4 min. The yield of Taxol from the HPLC chromatograms for *A. niger* and *F. solani* were about 173 and 55 μg/L, that being matched with the TLC analysis

Eight fungal endophytes were recovered from the leaves of *E. whitelockii* on PDA media, these isolates were morphologically identified based on their macroscopic and microscopic traits into four genera *Aspergillus*, *Penicillium*, *Cladosporium* and *Fusarium* (Fig. 1). Among the recovered genera, *Aspergillus* was represented by 55%, *A. niger*, *A. awomari*, *A. candidus, A. flavus* and *A. fumigatus.* Taxol productivity by the recovered endophytes of *E. whitelockii* were assessed on PDB, incubated at standard conditions, then Taxol was extracted and quantified. From the results (Fig. 1), the maximum Taxol yield was reported for *A. niger* (173.9 μg/L), followed by *Fusraium solani* (55.2 μg/L), *A. candidus* (40.2 μg/L) and *A. awomari* (35.2 μg/L). However, the other fungal isolates had a relatively low potency of Taxol productivity (2–10 μg/L). The putative sample spots gave the identical mobility rate and color of the authentic Taxol under UV-illumination at λ_254_ nm, were considered, ensuring the chemical identity of the samples as Taxol. The putative Taxol samples was scarped-off from the TLC silica, and dissolved in methanol. The concentration of the putative Taxol samples were confirmed by HPLC, gave a sharp peaks at 4.4 min that was identical to the authentic one. Thus, from the TLC and HPLC, the putative sample was relatively authenticated as Taxol.

### Identification of the most potent Taxol-producing fungi, and PCR mining of *bapt*

**Fig. 2:**
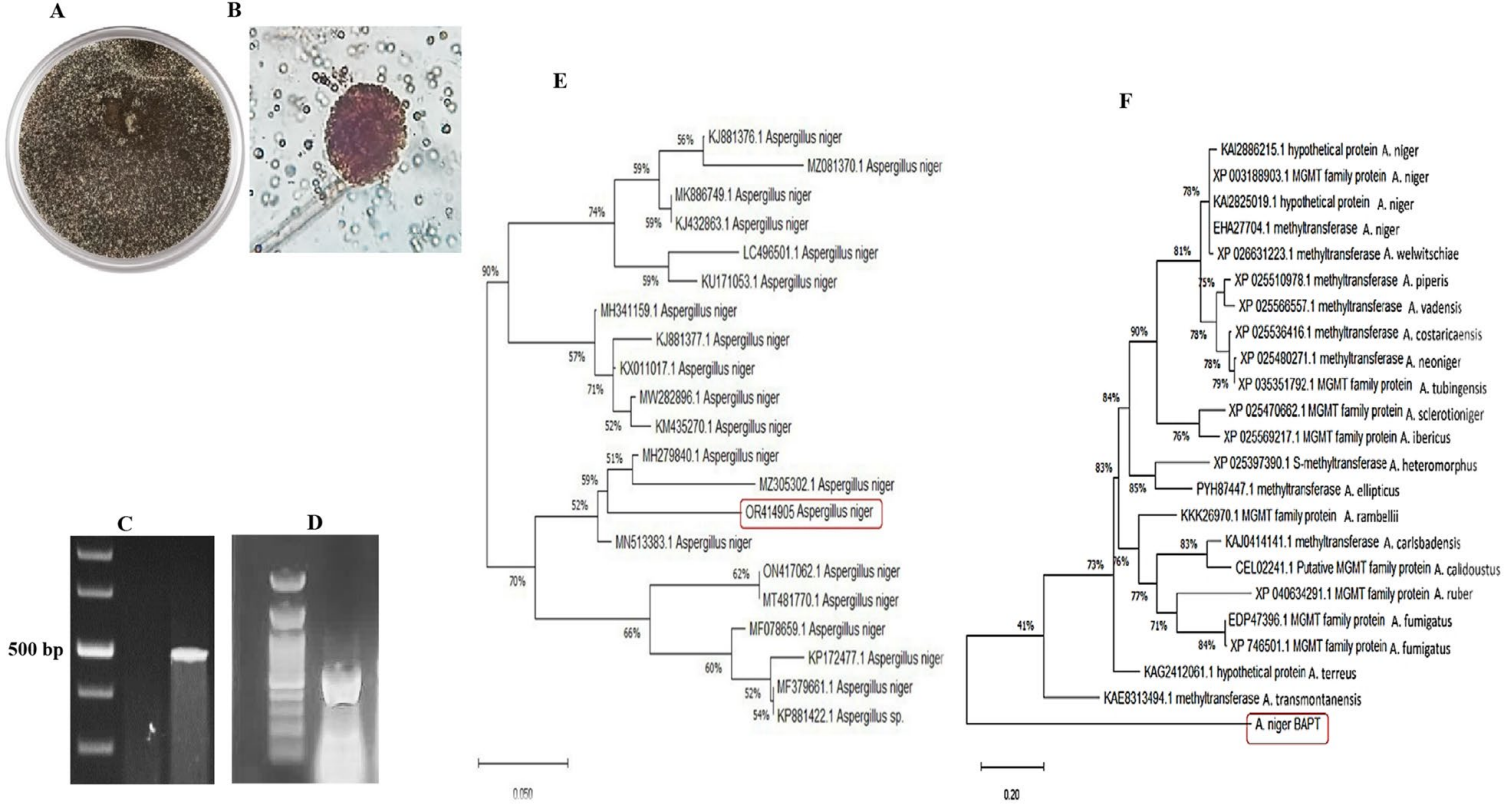
Morphological and molecular identification of the potent Taxol producing fungus. **A** Plate culture of A. niger on PDA medium after 6 days. **B** Conidial heads of *A. niger* with an oval shape, and biserriate strigma at 1000 × magnification. **C** PCR amplicon of the ITS region of *A. niger*. **D** PCR amplicon of *bapt* gene as Taxol rate-limiting gene of Taxol biosynthesis. **E** Phylogenetic relatedness of the ITS region of *A. niger* by the maximum neighbor-joining (NJ) model by the MEGA X software. **F** The phylogenetic relatedness of the sequence of putative protein sequence of the BAPT retrieved from translation of the *bapt* gene by the maximum neighbor-joining (NJ) model by the MEGA X. The DNA ladder was 1 kb (Nex-gene Ladder, Puregene, Cat.# PG010-55DI)

The morphological characteristics of the most potent Taxol-producing fungal endophyte of *E. whitelockii*, was observed by growing on PDA and Czapek’s-Dox agar media along for 10 days at 30 ℃, and their macro and microscopical traits were checked daily. The developed fungal colonies obviously appeared whitish, then turned to black after 4 days, the sides of the colonies appear pale yellow producing radiating fissures. The fungus has smooth colored conidiophores, radial biseriate conidial heads, with conidial size 3.5–5.0 mm and vesicle 45–80 μm (Fig. 2). The morphological identity of the current fungal isolate completely follow the descriptions of *A. niger* as described by Samson et al*.* [56] and Raper and Fennel, [39]

The morphological identity of the most potent isolate “*A. niger”* was verified from the molecular sequence of their rRNA region. The genomic DNA was extracted, used as a PCR template, and the resulting amplicon was ~ 500–520 bp (Fig. 2). The PCR product was purified, sequenced, and non-redundantly BLAST searched on Genbank. From the alignment analysis on the NCBI, the retrieved sequence was displayed 100% similarity with the isolates of *A. niger* with zero E-value. The sequence of ITS of *A. niger* EFBL-AG was deposited into the Genbank at accession # OR414905.1. The phylogenetic relatedness of *A. niger* was constructed by alignment with the data based deposited ITS sequences of *A. niger.* The isolate *A. niger* EFBL-AG OR414905.1, an endophyte of *E. whitelockii*, had a 99.5% identity with the isolates of *A. niger* with accession # MH341159.1, KP172477.1, MF078659.1, KJ881377.1, LC496501.1, KU171053.1, MW282896.1, MH279840.1, and KJ881376.1 with zero E-value and query coverage (98–100%).

The metabolic potency of Taxol biosynthesis of *A. niger* has been assessed based on the expression of 10-deacetyl-baccatin III-O-acetyltransferase, as a rate-limiting gene of Taxol biosynthesis. From the PCR results, an amplicon of *bapt* was ~ 450 bp for *A. niger*, the amplicon was sequenced (Fig. 2). The sequence of *bapt* was in silico translated by ExPASy portal tool (https://www.ebi.ac.uk/Tools/st/emboss_sixpack). The phylogenetic analysis of the BAPT protein was constructed by MEGA X software package (Fig. 2), displayed ~ 99.2% similarity with *A. niger* MGMT family proteins (XM025592527.1, XM003188855.1, XP040634291.1, CEL02241.1, KAJ04141.1, KKK26970.1, PYH87447.1, XP025390.1, XP025470662.1, XP025566557.1 and XP025510978), and 91% similarity with putative methyltransferase of *A. neoniger* (XM025626698.1, XP025536416.1, XP025566557.1, XP026631223.1). So, the results of TLC and HPLC analysis of Taxol were significantly complemented with PCR amplicons of *bapt*, as a rate-limiting gene of Taxol biosynthesis.

### Chemical identity of the extracted Taxol from *A. niger*

**Fig. 3:**
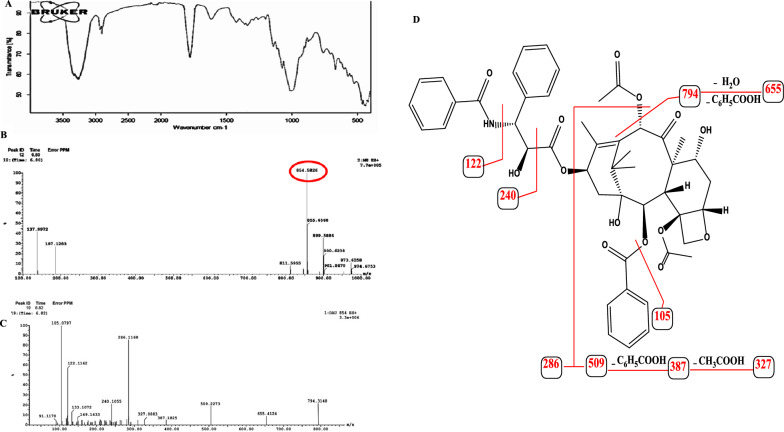
Chemical analysis of extracted Taxol from *A. niger*. **A** FT-IR spectrum of the extracted Taxol. From *A. niger*. **B** LC–MS chromatogram of the extracted Taxol. **C** LC–MS/MS fragmentation of the extracted Taxol. **D** Scheme of fragmentation pattern of Taxol by the 2nd MS

The chemical identity of Taxol extracted from *A. niger* was verified by UV–Vis, FT-IR, and LC–MS/MS Analyses. Based on the UV-absorption spectral analysis, the purified Taxol of *A. niger* exhibited a maximum absorption peak at 227 nm, that was consistent with the standard absorption spectrum of authentic Taxol. From the FT-IR spectra, the purified Taxol had a broad peak in the range of 3475.04 to 3290.39 cm^–1^ owing to the stretching of hydroxyl (OH) group and amide (-NH) group, as well as, to the stretching of the aliphatic CH groups in the area of 2976 to 2853 cm^–1^. The peaks at 1734.06 and 1601.65 were designated to the ester group and aromatic rings stretches, respectively. The COO stretching frequency peaked at 1259.69 cm^–1^, while the alkyl C-O stretching of ester appeared at 1073.10 cm^–1^. The Aromatic C and H bends frequency peaked at 1020.01 cm^–1^ as shown in Fig. 3.

The chemical structure of Taxol was resolved by UPLC-ESI–MS/MS in a positive mode. The LC–MS analysis revealed that the Taxol sample had a molecular mass to charge ratio of 854.5 m/z (Fig. 3), that was similar to authentic Taxol from *Taxus brevifolia* [57]. The molecular identity of Taxol has been further resolved from the LC–MS/MS analysis, the parent molecule of 854.5 m/z was further fragmented, displayed a similar fragmentation pattern of authentic Taxol [58]. The mass fragmentation of the parent Taxol molecule was illustrated in Fig. 3. The parent molecule of Taxol at *m/z* 854.5 [M + H]^+^ had MS/MS base peak ion at *m/z* 286 revealing the loss of Taxol side chain (C_16_H_15_NO_4_). The characteristic fragment ions were 749, 655, 509, 387, 327, 122 and 105 m/z were completely matched with the fragments of authentic Taxol, ensuring the chemical identity of putative sample of *A. niger* as Taxol.

### Antiproliferative and antifungal activity activities of ethyl acetate extract and purified Taxol from *A. niger*

**Fig. 4:**
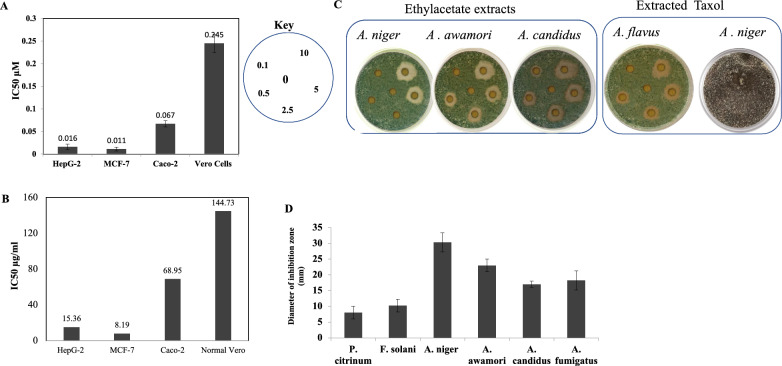
The antiproliferative and antifungal activities of the extracted Taxol and crude ethylacetate extracts of *A. niger.* A, The activity of the putative Taxol **A**, and crude ethylacetate extracts (**B**) towards the tumor cell lines HepG-2, MCF-7, Caco-2, compared to the normal Vero cells, a revealed from the IC_50_ values. **C** The antifungal activity of the extracted Taxol and crude ethylacetate extracts against *A. flavus* at concentrations 0.1, 0.5, 2.5, 5 and 10 μg/mL. **D** The diameter of the inhibition zones of the tested fungal extracts against* A. flavus*

The antiproliferative activity of ethyl acetate extract and purified Taxol from *A. niger* was evaluated against human hepatocellular (Hep-G2), breast (MCF-7) and intestinal (Caco-2) carcinoma cells, compared to the normal Vero cells. The fungus was grown on PDB medium, incubated at the desired conditions, the culture filtrate was extracted with ethyl acetate, and Taxol was fractionated by TLC. The putative Taxol spots with the same mobility rate and color of the authentic one were scraped off and Taxol was eluted, and their activity was evaluated against the experimental cancer cell lines. From the viability of cells (Fig. 4), the ethyl acetate extract of *A. niger* showed a significant effect against MCF-7 (IC_50_ value 8.9 μg/mL), Hep-G2 (IC_50_ value 15.36 µg/mL) and Caco-2 (IC_50_ value 68.9 μg/mL), compared to the normal Vero cells. The selectivity index of the ethyl acetate extracts of *A. niger* towards MCF-7 and HepG-2 cells, were 17.6 and 9.4, respectively. The purified Taxol of *A. niger* exhibited a significant cytotoxic activity towards the tested cell lines. From the IC_50_ values, Taxol had a strong activity against the MCF-7 and HepG2 cells (~ 0.014 μM), and Caco-2 cells (0.067 μM), with selectivity index about 22.2, revealing the efficiency and specificity of Taxol in targeting the tumor cells than normal Vero cells.

The antifungal activity of the ethyl acetate extracts of the recovered fungal isolates from *E. whitelockii* was assessed by well-diffusion assay, towards *Aspergillus flavus*, as a model human fungal pathogen. From the data (Fig. 4), obviously the positive Taxol-producing endophytic fungal isolates have a remarkable activity against *A. flavus,* in a concentration-dependent manner. The ethyl acetate extracts of *A. niger* had the highest activity against *A. flavus,* as revealed from the diameter of the inhibition zone (30 mm) followed by *A. awamori* (23 mm), and *A. candidus* and *A. fumigatus* (17–18 mm) at 10 μg/mL. Practically, the putative Taxol sample exhibited a substantial activity against *A. flavus* in a concentration-dependent manner, in contrast to the absence of such activity against *A. niger* as a producer strain (Fig. 4). The lack of biological activity of the extracted Taxol on *A. niger*, confirm the possessing of a specific resistance mechanism to Taxol that might be by blocking the receptors on the surface of cell membrane or by re-orienting the molecular stereo-structure of tubulin proteins, to be inaccessible for binding with Taxol.

### Anti-Wound healing activity of the cells due to the purified Taxol of *A. niger*

**Fig. 5:**
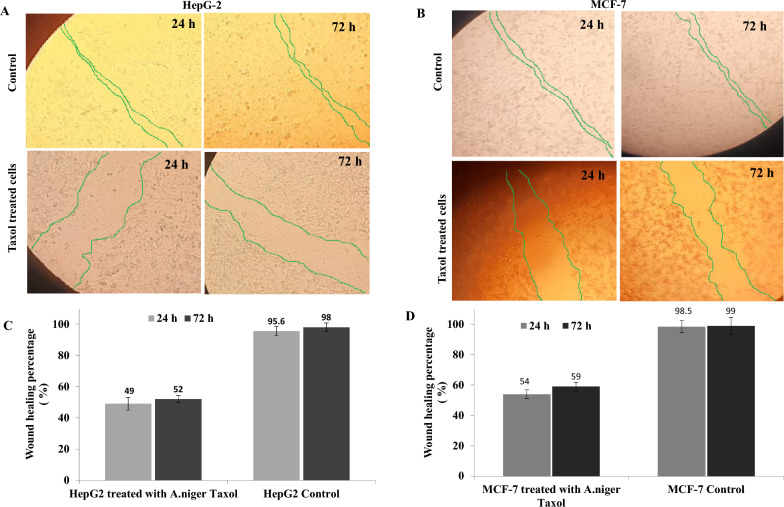
Wound healing assay of the HepG2 and MCF-7 cells in response to the purified Taxol of *A. niger* comparing to the untreated cell lines (negative control), after 24 ad 72 h. After 24 h of growth of the cells as homogenous monolayer, a scratch was made and the extracted Taxol was added (IC50 values 0.02 μM), to the medium, then the wound healing was measured at zero time and after 24 h and 72 h of incubation for the cell lines HepG-2 (**A**) and MCF-7 (**B**). The percentage of wound healing of the HepG-2 (**C**) and MCF7 (**D**) cells in response to Taxol treatment

The effect of purified Taxol of *A. niger* on the migration of the HepG2 and MCF-7 cells, was assessed by measuring the wound closure after 24 and 72 h, compared to the untreated cells. The results showed that the wound closure of HepG-2 and MCF-7 monolayer cells was remarkably suppressed by the purified Taxol*,* compared to the negative control (Fig. 5). The wound healing percentage of the homogenous monolayer of HepG2 and MCF-7 was approximated by 49% and 54%, after 24 h, and by 52% and 59% after 72 h, respectively, compared to the untreated cells. The suppression of wound healing of HepG2 and MCF-7 cells upon addition of the purified Taxol of *A. niger*, confirmed the interference with the cellular regeneration, and matrix formation of tumor cells, ultimately halts their metastasis.

### Apoptosis and cell cycle analysis of the MCF7 responsive to the purified Taxol of *A. niger*

**Fig. 6:**
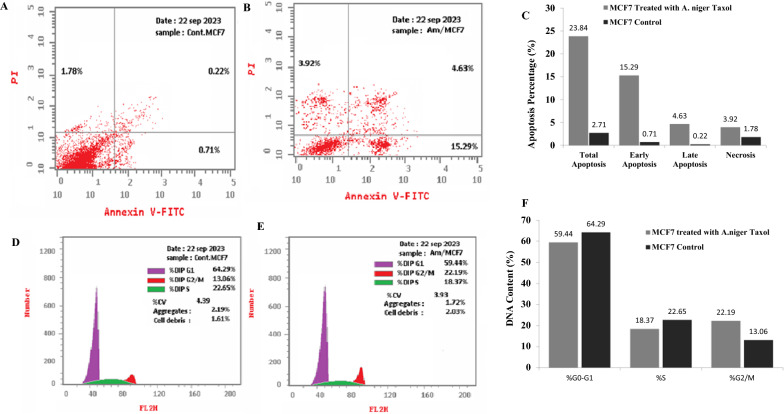
Flow cytometric apoptotic analysis of the MCF-7 cells by Annexin V-FITC. The cells were exposed to IC25 concentration of Taxol, the apoptosis was measured after 48 h of incubation. Apoptotic analysis of MCF-7 -cells without treatment (**A**), and in response to treatment with Taxol (**B**), and the overall quantitative results of apoptosis (**C**). The cell cycle analysis of the MCF-7 without treatment (**D**), and in response to treatment with Taxol (**E**) and overall quantitative analysis of cell cycle (**F**)

The effect of the purified Taxol of *A. niger* on the apoptosis of MCF-7 cells was assessed by Annexin V-propidium iodide assay. From the results (Fig. 6), a significant shift was observed for the cells to an early apoptotic and late apoptotic stages, due to Taxol of *A. niger,* compared to the control cells*.* The MCF-7 cells percentage in early, late apoptosis, and necrosis were about 15.2%, 4.6% and 3.9%, in response to Taxol of *A. niger,* while, the early apoptosis, late apoptosis and necrosis were represented by about 2.71, 0.71 and 1.78%, respectively, in control cells. The percentage of total apoptosis of the MCF-7 cells was increased by about 11 folds in response to Taxol treatment, compared to the control cells.

The MCF-7 cell cycle with Taxol treatment was analyzed by propidium iodide, the cells were amended with purified Taxol of *A. niger,* at their IC_25_ values, after incubation, the cells were harvested by centrifugation, fixed by 1 mL ice cold 70% ethanol for 2 h at 4 ℃. The DNA content of the cells was analyzed and the ratios of G0-G1, S and G2-M cells were determined. From the results (Fig. 6), the maximum growth arrest of MCF-7 cells in response to Taxol treatment was reported at the G2/M values, compared to the control cells. However, the purified Taxol had no obvious effect on the S-phase and G0-G1phases of MCF-7, compared to the negative control cells. Overall, the purified Taxol from *A. niger* had a significant arresting effect to the cell cycle at G2/M phase.

### Bioprocess optimization of Taxol production of *A. niger* by the plackett–burman and central composite designs (CCD)

**Fig. 7:**
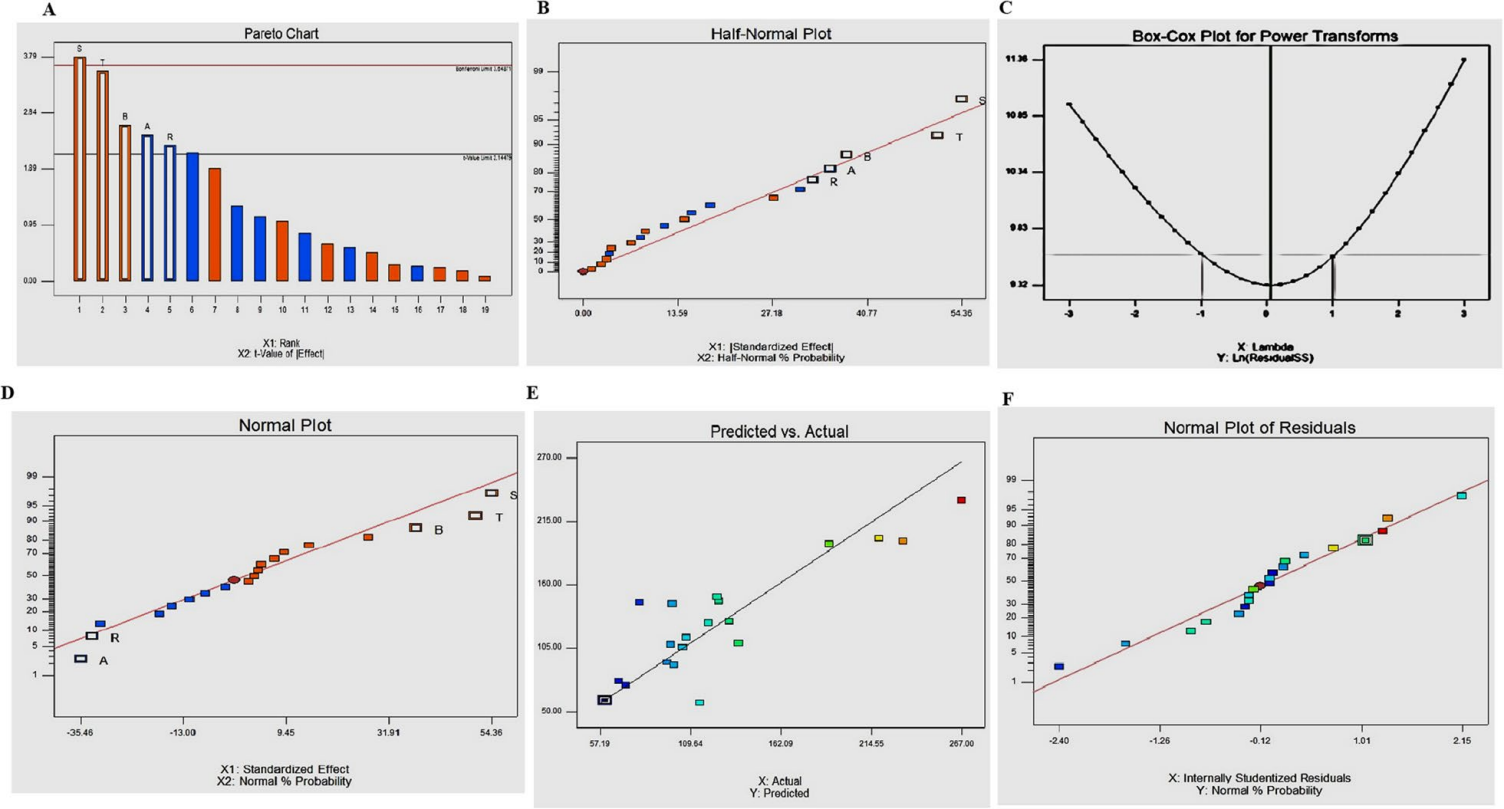
The impacts of several variables on Taxol production corresponding to the Plackett–Burman experimental design. The normal probability plots of the variables for Taxol production by *A. niger* as determined by the first order polynomial equation. **A**: The Pareto chart displays the significance of all variables. The half -Normal plot (**B**), The Box-Cox power transform (**C**), and the normal plot of the standardized effect with normal possibility (**D**). **E** Plot of correlation between predicted and actual Taxol yield of. *A. niger.* **F** Plot of the standardized effect with normal residuals

The productivity of Taxol by *A. niger,* an endophyte of *E. whitelockii,* was maximized by optimizing the fungal nutritional requirements. The medium constituents and their interactions are crucial in regulating the biosynthetic machinery of fungal secondary bioactive metabolites. The Response Surface Methodology has been frequently employed to nutritionally optimize the fungal growth to maximize their secondary metabolites yield [44]. Nineteen variables including carbon, nitrogen precursors, growth modulators, and elicitors were evaluated. The lowest and highest values of Plackett–Burman design of each parameter were summarized in (Table 1). After fungal growth, Taxol was extracted, quantified, and the significance of the independent variables affecting Taxol productivity by *A. niger*, along with the predicted and actual responses from the matrix of Plackett–Burman design were summarized (Table 2). All experiments were conducted in triplicate. Statistical analysis of this design illustrated the significance of this Model with F-value of 9.04. There is only 0.05% noise of this as revealed from the F-Value. Also, the model terms hold significant importance when the values of "Prob > F" are less than 0.0500. The "Predicted R-Squared" of 0.5174 is in rational agreement with the "Adj. R- Squared" of 0.6791. "Adeq. Precision" signal to noise ratio was about 10.011 revealing the adequacy of the signal (Table 3). The plotting of the main effects and normal probability of the tested variables were assessed (Fig. 7), demonstrating that there were five different independent variables, S-methyl jasmonate (X15), T- Ammonium sulfate (X14), B-Lactose (X4), A-Maltose (X10) and R-Fluconazole (X13), had a significant effect on Taxol productivity by *A. niger*. The significance of the factors on Taxol productivity by *A. niger*, as well as the actual and predicted yield of Taxol, can be observed through the Pareto chart and probability plots of the independent variables (Fig. 7). The evaluation of statistical significance of each variable was conducted using the p-value and student’s t-test, as shown in Table 3. The distribution of residuals around the diagonal line confirmed the independent normality of the variables, indicating a precise alignment between the predicted and actual Taxol yield. The initial polynomial equation for Taxol production by *A. niger* reflecting the significant independent variables was represented by the following:

**Table 1:** Lowest and highest values of the selected parameters in Plackett–Burman Design for optimization of Taxol production

No	Variables (*g*/L)	Value	
		Low (− 1)	High (+ 1)
X1	Maltose	2.0	6.0
X2	Lactose	2.0	6.0
X3	Sucrose	2.0	6.0
X4	Peptone	3.0	8.0
X5	Soy tone	3.0	8.0
X6	Yeast extract	3.0	6.0
X7	Ammonium tartrate	2.0	4.0
X8	Sodium acetate	2.0	4.0
X9	Cysteine	3.0	6.0
X10	Phenyl alanine	1.0	3.0
X11	Methionine	2.0	4.0
X12	Glycine	2.0	4.0
X13	Sodium nitrate	1.0	3.0
X14	Calcium chloride	1.0	3.0
X15	Magnesium sulphate	0.5	2.0
X16	Potassium hydrogen phosphate	1.0	4.0
X17	Fluconazole	1.0	3.0
X18	Methyl jasmonate	0.1	1.0
X19	Ammonium sulphate	2.0	5.0

**Table 2:** The Plackett–Burman experimental design matrix for Taxol production by *A. niger*

Run	X1	X2	X3	X4	X5	X6	X7	X8	X9	X10	X11	X12	X13	X14	X15	X16	X17	X18	X19	Actual Taxol yield (µg/L)	Predicted Taxol yield (µg/L)	Residuals
1.	1	− 1	− 1	− 1	1	1	1	− 1	1	1	− 1	− 1	1	1	1	1	1	1	− 1	137.51	109.32	28.19
2.	1	1	1	1	− 1	1	− 1	1	− 1	− 1	− 1	− 1	1	1	− 1	1	1	− 1	− 1	59.69	59.20	0.49
3.	− 1	− 1	− 1	− 1	1	1	− 1	1	1	− 1	− 1	1	1	1	1	− 1	1	− 1	1	98.10	108.36	− 10.26
4.	1	− 1	1	− 1	1	− 1	− 1	− 1	− 1	1	1	− 1	1	1	− 1	− 1	1	1	1	120.30	126.02	− 5.72
5.	1	− 1	− 1	− 1	− 1	− 1	− 1	1	1	− 1	1	1	− 1	− 1	1	1	1	1	− 1	68.42	76.55	− 8.13
6.	1	1	1	1	− 1	1	− 1	− 1	− 1	− 1	1	1	− 1	1	1	− 1	− 1	1	1	195.50	167.35	28.15
7.	**− 1**	**1**	**1**	**− 1**	**− 1**	**1**	**1**	**1**	**1**	**− 1**	**1**	**− 1**	**1**	**− 1**	**− 1**	**− 1**	**− 1**	**1**	**1**	**267.16**	**233.21**	**33.95**
8.	− 1	− 1	1	1	− 1	1	1	− 1	− 1	1	1	1	1	− 1	1	− 1	1	− 1	− 1	115.22	57.85	57.37
9.	1	− 1	− 1	1	1	1	1	− 1	1	− 1	1	− 1	− 1	− 1	− 1	1	1	− 1	1	72.26	72.00	0.26
10.	1	1	− 1	1	1	− 1	− 1	1	1	1	1	− 1	1	− 1	1	− 1	− 1	− 1	− 1	69.76	92.36	− 22.6
11.	1	1	1	− 1	1	− 1	1	− 1	− 1	− 1	− 1	1	1	− 1	1	1	− 1	− 1	1	99.42	143.45	− 44.03
12.	1	1	− 1	− 1	1	1	1	1	− 1	1	− 1	1	− 1	− 1	− 1	− 1	1	1	− 1	107.13	113.29	− 6.16
13.	− 1	1	− 1	− 1	− 1	− 1	1	1	− 1	1	1	− 1	− 1	1	1	1	1	− 1	1	126.56	145.22	− 18.66
14.	− 1	− 1	1	1	1	1	− 1	1	− 1	1	− 1	− 1	− 1	− 1	1	1	− 1	1	1	190.75	195.03	− 4.28
15.	1	− 1	1	1	− 1	− 1	1	1	1	1	− 1	1	− 1	1	− 1	− 1	− 1	− 1	1	105.72	105.23	0.49
16.	− 1	1	− 1	1	− 1	− 1	− 1	− 1	1	1	− 1	1	1	− 1	− 1	1	1	1	1	219.88	200.00	19.88
17.	− 1	1	1	1	1	– 1	1	− 1	1	− 1	− 1	− 1	− 1	1	1	− 1	1	1	− 1	125.24	149.25	− 24.01
18.	− 1	1	1	− 1	1	1	− 1	− 1	1	1	1	1	− 1	1	− 1	1	− 1	− 1	− 1	132.46	127.02	5.44
19.	− 1	− 1	− 1	1	1	− 1	1	1	– 1	− 1	1	1	1	1	− 1	1	− 1	1	− 1	99.46	144.25	− 44.79
20.	− 1	− 1	− 1	− 1	− 1	− 1	− 1	− 1	− 1	− 1	− 1	− 1	1	− 1	− 1	− 1	− 1	− 1	− 1	100.19	90.23	9.96

**Table 3:** Analysis of regression statistics and variance ANOVA for Placket-Burman design

Source	Sum of squares	Df	Mean Square	F Value	p-Value Prob > F	
Model	46,586.94	5	9317.39	9.04	0.0005	Significant
A-Maltose	6287.06	1	6287.06	6.1	0.027	
B-Lactose	7159.33	1	7159.33	6.95	0.0196	
R-Fluconazole	5431.81	1	5431.81	5.27	0.0376	
S-Methyl jasmonate	14,775.05	1	14,775.05	14.34	0.002	
T-Ammonium sulfate	12,933.7	1	12,933.7	12.55	0.0032	
Residual	14,427.3	14	1030.52			
Cor Total	61,014.24	19				

	Coefficient		Standard Error	95% CI		VIF
Factor	Estimate	Df		Low	High	
Intercept	127.47	1	7.178166797	112.074363	142.8656366	
A-Maltose	− 17.73	1	7.178166797	− 33.125636	− 2.33436342	1
B-Lactose	18.92	1	7.178166797	3.52436342	34.31563658	1
R-Fluconazole	− 16.48	1	7.178166797	− 31.8756365	− 1.08436342	1
S-Methyl jasmonate	27.18	1	7.178166797	11.78436342	42.57563658	1
T-ammonium sulfate	25.43	1	7.178166797	10.03436342	40.82563658	1

Taxol productivity = 65.49333 − 8.865 ∗ Maltose + 9.46 ∗ Lactose − 16.48 ∗ Fluconazole + 60.4 ∗ Methyl jasmonate + 16.95333* Ammonium sulphate.

The highest actual (267.16 µg/L) and predicted (233.5 µg/L) yields of Taxol by *A. niger* was reported at Run #7, with the Plackett–Burman design, with about 1.5 folds increases in Taxol yield of *A. niger*, compared to the control “non-optimized” fungal cultures (173.9 µg/L).

The most significant parameters affecting Taxol production by *A. niger* as revealed from the Plackett–Burman design including maltose, lactose, fluconazole, methyl-jasmonete and ammonium sulfate were further optimized by Central Composite Design (CCD). The interactions of the five parameters “maltose, lactose, fluconazole, methyl-jasmonate and ammonium sulfate” were tested at five levels, to assess their physiological interactions of on Taxol production *A. niger*. From the CCD results (Table 4), the maximum productivity of Taxol (279.8 µg/L) by *A. niger* with the CCD bioprocessing was recorded at the run #7, i.e. by about 1.1 folds increments over the Plackett–Burman deisgn. The highest Taxol productivity by *A. niger* upon CCD, was achieved at 4 *g*/l maltose, 4 *g*/L lactose, 1.0 *g*/L fluconazole, 0.2 *g*/l methyl-jasmonate and 2 *g*/L ammonium sulfate incubated for 15 days. The factors with p-value < 0.1 were considered to be significant. The most significant factors affecting Taxol production was reported at 90% confidence, with r2 value 97.8%, revealing the goodness of fit of the regression model. Thus, the interaction between maltose, lactose, fluconazole, and methyl-jasmonate were the most significant factors affecting Taxol production by *A. niger*.

**Table 4:** Matrix and responses of the CCD for the significant factors affecting Taxol production by *A. niger*

	Maltose (*g*/L)	Lactose (*g*/L)	Fluconazole (*g*/L)	Methyl jasmonate (*g*/L)	Ammonium sulfate (*g*/L)	Taxol yield (µg/L)
1	2	8	0.1	0.2	2	152
2	1	4	0.5	0.1	5	82.2
3	2	2	0.5	0.1	3	110.2
4	1	8	1	0.4	4	162.2
5	8	12	4	0.4	1	93.2
6	2	8	2	0.6	2	213.8
7	4	4	2	0.6	3	279.8
8	8	8	2	0.4	2	130.1
9	2	12	2	0.2	4	93.4
10	4	4	4	0.2	3	84.1
11	8	4	4	0.4	2	120.1
12	1	4	4	0.8	4	132.1
13	8	8	2	0.8	5	149.7
14	4	2	2	0.1	5	210.1
15	1	2	4	0.1	1	135.9
16	4	2	4	0.6	1	230.1
17	4	8	2	0.8	2	161.6
18	4	2	0.5	0.2	2	141.1
19	8	4	0.5	0.6	4	102.6
20	1	4	0.5	0.6	3	120.8
21	12	4	0.1	0.8	2	120.2
22	8	4	0.1	0.6	4	160.8
23	4	8	2	0.4	5	164.9
24	4	1	2	0.6	4	126.7
25	12	8	4	0.8	5	137.7
26	12	12	2	0.1	3	87.9
27	12	12	4	0.1	3	210.3
28	8	1	4	0.1	3	203.9
29	8	12	4	0.2	4	128.9
30	4	1	4	0.2	4	89.0
31	4	8	2	0.6	1	84.9

### Metabolic stability of Taxol production by *A. niger* with the storage and effect of the plant leaf extracts on restoring the Taxol productivity

**Fig. 8:**
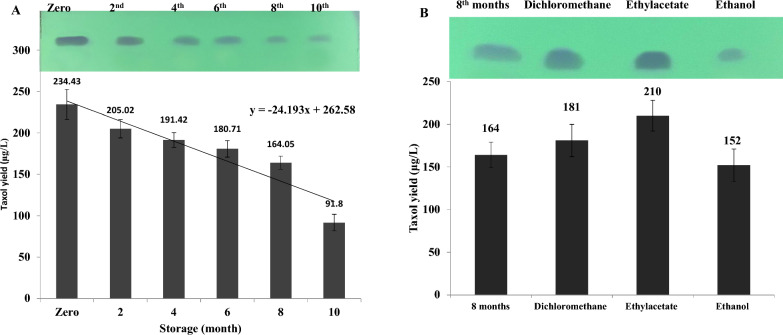
Biosynthetic stability of Taxol by *A. niger* in response to storage and effect of plant extracts on restoring its biosynthetic Taxol machinery. **A** The productivity of Taxol for *A. niger* in response to fungal preservation as slope culture (The upper panel is the TLC, the lower panel is the yield of Taxol). **B** The yield of Taxol by *A. niger* in response to amendment with different extracts of *E. whitelockii* (The upper panel is the TLC, the lower panel is the yield of Taxol)

The influence of storage of *A. niger* at 4 ℃ along 10 months was assessed on their Taxol biosynthetic potency, compared to the zero culture. After incubation of each culture, Taxol was extracted and quantified by TLC and HPLC. A noticeable relative stability of Taxol biosynthetic machinery has been observed till 6 months of storage as slope cultures at 4 ℃ (Fig. 8). The productivity of Taxol by *A. niger* was attenuated by about 1.25 folds after the 6 months (180.7 μg/L) of storage, compared to the first culture (234.43 μg/L). The yield of Taxol by *A. niger* was reduced by about 2.6 folds at the 10th month of storage (91.8 μg/L). Thus, from the linear equation of Taxol productivity by *A. niger*, the biological half-life time of metabolic biosynthetic stability of Taxol was approximated by about 6.1 months as slope cultures at 4 ℃. As well as, the mycelial melanin pigmentation of *A. niger* was obviously faded with the fungal storage, revealing the apparent connection with the expression of the biosynthetic genes of polyketides with the storage time, that might be correlated with the Taxol reduction over the time [21].

The effect of organic leaves extracts of *E. whitelockii* on restoring the Taxol biosynthetic potency of *A. niger* has been assessed. The extracts of dichloromethane, ethyl acetate and ethanol of *E. whitelockii* leaves, was amended to 5 days pre-culture of the zero culture and 8 months stored *A. niger*, then completed to 15 days of incubation, Taxol was extracted and quantified. From the obtained results (Fig. 8), the ethyl acetate extracts of *E. whitelockii* displayed a slight inducing effect of Taxol biosynthesis of 8 months stored culture of *A. niger* by about 1.3 folds (210 μg/L), however, this extract had no obvious effect on Taxol production by the zero culture of *A. niger*. The extracts of dichloromethane and ethanol had no noticeable effect on induction of Taxol biosynthesis by the stored *A. niger* culture. The remarkable inducing effect of ethyl acetate extracts, compared to other extracts, might be due to the high polarity of the ethyl acetate extracting unique compounds, than the other solvents.

## Discussion

Despite the fungal fast growth, resistance to mechanical stress, and feasibility of genetic manipulation, the commercial production of Taxol by fungi has been halted by the rapid loss of Taxol productivity with the storage and subculturing [14, 18, 22, 59]. Thus, screening for a novel endophytic fungal isolate inhabiting the plants of ethnopharmacological uses, could have relatively stable Taxol-producing machinery. From literature, the biological and metabolic identities of the fungal endophytes inhabiting *E. whitelockii* remains equivocal, thus, mining of fungal endophyte with metabolically stable Taxol-producing machinery with the storage was the main objective of this study. Eight fungal isolates were recovered from the leaves of *E. whitelockii,* the highest Taxol producing potency was reported for *A. niger* (173.9 μg/L). The isolate of *A. niger* was morphologically identified based on the descriptions of Samson et al*.* [56] and Rapper ad Fennel et al*.* [39]. The isolate of *A. niger* was molecularly identified based on its internal transcribed spacer (ITS) sequence, the sequence was deposited on Genbank with accession number OR414905.1. This is the first report describing isolate of *A. niger as* an endophyte of *E. whitelockii*, that has the Taxol producing potency. Similar results reported the Taxol producing potency from gymnosperms; *Ginkgo biloba* [44] and *Podocarpus gracilior* [21]. The Taxol yield by *A. niger* has been matched with *Penicillium polonicum* [44], *A. flavipes* [60], *A. terreus* [21], endophytes of *Podocarpus gracilior*, as well as endophytes of *Taxus* sp such as *A. niger* [61], *Fusarium solani* [62] and *A. candidus* [63]. The metabolic potency of Taxol production was verified by the PCR mining of 10-deacetyl-baccatin III-O-acetyltransferase as rate-limiting gene of Taxol biosynthesis. Molecular verification of Taxol biosynthesis via PCR mining of the signature genes encoding the rate-limiting enzymes was recognized as preliminary marker for assessing the molecular blueprint of Taxol biosynthesis [21, 64, 65].

The chemical identity of the purified Taxol from *A. niger* was confirmed by HPLC, UV-absorption spectra, FT-IR spectra, and LC–MS/MS. Collectively, from these spectral and chromatographic analysis, the purified putative Taxol from *A. niger* had the same spectral and chemical structures of the authentic Taxol from *T. brevifolia* [14]. Additionally, the putative Taxol sample from *A. niger* had a molecular mass to charge ratio of 854.5 m/z, which was identical to the authentic Taxol from *T. brevifolia* [14]. Furthermore, the parent molecule of the putative Taxol from *A. niger* exhibited the same molecular fragmentation pattern as the authentic Taxol, confirming their chemical identity as Taxol [20, 58, 66].

The antiproliferative activity of ethyl acetate extract and Taxol from *A. niger* was evaluated against human hepatocellular (Hep-G2), breast (MCF-7) and intestinal (Caco-2) carcinoma cells compared to the Vero cells. From the IC_50_ values, the ethyl acetate extract of *A. niger* had a significant effect against MCF-7 (8.9 μg/mL), Hep-G2 (15.36 µg/mL), with obviously tiny activity to the Vero cells. The purified Taxol of *A. niger* had a significant activity towards the MCF-7 and HepG2 cells (~ 0.014 μM), and Caco-2 cells (0.067 μM), with selectivity index 22.2, revealing the efficiency and specificity of Taxol in targeting the tumor cells than normal ones. According to the National Cancer Institute (NCI) reports, the potent cytotoxic compounds should be of IC_50_ value < 20 μg/mL [67]. Ethyl acetate extract was found to have a potent cytotoxic activity with IC_50_ of < 20 μg/mL on MCF-7 and Hep-G2, that being similar with the ethyl acetate extracts of *A. flavus,* with IC_50_ for MCF-7 cells of 16.25 µg/mL [68].

*A. flavus* is the second most prevalent opportunistic pathogen that causes both invasive and superficial infections in humans, exhibiting the highest levels of morbidity and mortality [69]. Ethyl acetate extract of *A. niger* of *E. whitelockii* exhibited superior antifungal activity than ethyl acetate extract of *A. niger,* an endophyte of *Alangium salviifolium,* or other host plants against *A. flavus* [70, 71]. This finding could be attributed to the fluctuation in the metabolite contents of fungal endophytes in relation to different host plants [22]. Taxol purified from *A. niger* has superior antiproliferative activity to Hep-G2 and MCF-7 tumor cells than *P. polonicum* Taxol (IC_50_ values of 4.06 μM and 6.07 μM) [44]. The antiproliferative activity of *A. niger* extracted Taxol towards the various human carcinoma cell lines were closely similar to Taxol purified from various fungal sources [20, 72–75]. Taxol inhibits the formation of functional spindles during metaphase in mitosis and cellular proliferation, disrupting the normal reorganization of the microtubules, ultimately leads to the induction of apoptosis and cell mortality during the G2/M phase of cell cycle [76–78]. Obviously, the Taxol-producing endophytic fungi have a remarkable activity against *A. flavus,* in a concentration-dependent manner. The putative Taxol sample has a significant activity against *A. flavus* in a concentration dependent manner. The lack of activity of the extracted Taxol on *A. niger*, the same Taxol-producing fungal isolate, confirm the possessing of a specific resistance mechanism to Taxol that might be by blocking the receptors on the cell membrane or re-orienting the molecular stereo-structure of tubulins, to be inaccessible for binding with Taxol. Similar results for dual antiproliferative and antifungal activities of Taxol were reported [79].

The effect of purified Taxol of *A. niger* on the migration of the HepG2 and MCF-7 cells, was evaluated by measuring the wound closure. The wound healing of the HepG2 and MCF-7 was estimated by 71% and 79% after 72 h, compared to the control. The plausible suppression of healing of tumor cells with the purified *A. niger* Taxol, confirmed the interference with the cellular divisions, and matrix formation, with ultimate halting of their metastasis. Taxol was extensively researched for its ability to act as an anti-angiogenic agent, hinder wound healing, and impede cell migration across various cell lines [80, 81]. Taxol inhibited the human umbilical vein endothelial cells migration, decreasing the length of microtubules; reducing the peripheral microtubules [80]. The effect of the purified Taxol of *A. niger* on the apoptosis of MCF-7 cells was assessed, a significant shift has been observed for the normal cells to apoptotic phase, responsive to Taxol*,* compared to control*.* The percentage of total apoptosis of the MCF-7 cells was increased by about 11 folds in response to *A. niger* Taxol, compared to the control cells. The cell cycle of MCF-7 in response to Taxol treatment was analyzed with the purified Taxol at IC_25_ values. The maximum growth arrest of MCF-7 cells in response to Taxol was reported at the G2/M values, compared to the control cells, that being similar with previous results [82]. The same biological effect of *A. niger* Taxol on cell cycle, antiproliferative activity, and cell migration of tumor cells with the Taxol from *T. brevfolia*, ensures the identical molecular stereo-structure, orientation and structure activity relationships of the purified sample with the authentic one.

The productivity of Taxol by *A. niger,* endophyte of *E. whitelockii,* was maximized by the nutritional optimization with the response surface methodology. The significant variables affecting Taxol productivity by *A. niger* was methyl jasmonate, ammonium sulfate, lactose, maltose and fluconazole. The actual yield of Taxol by *A. niger* was increased by ~ 2.3 folds, compared to the control cultures. Similar paradigm of maximizing the yield of bioactive metabolites by fungi via nutritional optimization with the Plackett–Burman Design was reported, considering the interactions of the components in regulating the biosynthetic machinery of secondary metabolites in fungi [17, 44, 50, 66]. Similar optimization protocol has been employed for maximizing to the Taxol productivity by *P. polonicum* and *A. terreus* [21, 44]. Methyl jasmonate has been recognized a significant elicitor triggering the crosstalk of the plasma membrane receptors, stimulating the plant defense responses, by generating the reactive oxygen species, induce the overexpression of the secondary metabolites [83]. Actually, the Taxol yield by *A. niger*, an endophyte of *E. whitelockii,* was similar to those of *A. terreus*, endophyte of *Podocarpus gracilior* (265.4 μg/L) [20, 21], *A. niger*, endophyte of *Taxus cuspidata* (273 μg/L) [61], *A. candidus*, endophyte of *Taxus media* (112 μg/L) [15], and *A. fumigatus*, endophyte of *Podocarpus* sp [3]. However, the Taxol yield by the current isolate of *A. niger* was higher than those reported by *F. proliferatum* (240 ng/L) and *Colletotrichum gloeosporioides* (120 ng/L), endophytes of *Taxus cuspidata* [63].

The Taxol productivity of *A. niger* preserved as slope culture at 4 ℃ along 10 months was assessed, a relative stability of Taxol biosynthetic machinery has been observed till 6 months. The Taxol productivity by *A. niger* was attenuated by ~ 1.25 folds after 6 months of storage as slope culture, compared to the first culture, thus, the biological half-life time of metabolic biosynthetic stability of Taxol was approximated by 6.1 months as slope culture. Attenuation of the Taxol productivity by fungi has been reported as one of the most challenges that halt the further implementation of fungi to be an industrial platform for Taxol production [4, 17, 18, 20, 21, 44]. Similarly, the Taxol biosynthetic stability by *A. flavipes* was reduced by 2 folds by the 6th month storage (135 μg/L) [16]. The yield of Taxol by *A. terreus* was drastically reduced by 4 folds (78.2 μg/L) by the 6th month of storage, compared to the 1st culture [20, 21], that being lower than the biosynthetic stability of Taxol by current isolate of *A. niger,* at the same conditions (174 μg/L)*.*

The ethyl acetate extracts of *E. whitelockii* displayed a slight inducing effect on Taxol biosynthesis of 8 months stored culture of *A. niger* by about 1.3 folds (210 μg/L). The remarkable inducing effect of ethyl acetate extracts might be attributed to the higher polarity of the ethyl acetate extracting unique compounds, than the other solvents. So, the Taxol biosynthetic machinery of *A. niger* may be influenced by chemical signals from the host plant or its associated microbial flora [15, 20].

In conclusion, *A. niger,* an endophyte of *E. whitelockii,* had the most promising yield of Taxol with relative metabolic stability till the 6th month of storage. The chemical identity of purified *A. niger* Taxol was confirmed by the FT-IR, HPLC and LC–MS/MS analysis, compared to the authentic one. The purified Taxol of *A. niger* displayed a significant antiproliferative activity, strong inhibitory effect on tumor cells migration, arresting the cell cycle at the G2/M phase. The biological half-life time of Taxol productivity by *A. niger* was about 6 months as slope culture at 4 ℃, with partial restoring to the metabolic potency of Taxol productivity by *A. niger* upon addition of ethyl acetate plant extract, ensuring possessing of some plant-derived signals that triggers the cryptic Taxol encoding genes. Metabolic characterization of the chemical identity of the plant-derived signals, and their effect on restoring the molecular expression of the Taxol biosynthetic machinery by the fungus, are the ongoing studies by our lab.

## Data Availability

All datasets generated for this study are included in the manuscript.

## References

[1] Hamed MM, Mohamed MA-E-M, Ibrahim MT (2015). Phytochemical investigation and cytotoxic characterization of bioactive constituents from Conyza dioscoridis. Planta Med.

[2] Wani MC, Taylor HL, Wall ME, Coggon P, McPhail AT (1971). Plant antitumor agents. VI. Isolation and structure of Taxol, a novel antileukemic and antitumor agent from Taxus brevifolia. J Am Chem Soc.

[3] Sun D, Ran X, Wang J (2008). Isolation and identification of a Taxol-producing endophytic fungus from Podocarpus. Acta Microbiol Sin.

[4] El-Sayed AS, Fathalla M, Yassin MA, Zein N, Morsy S, Sitohy M, Sitohy B (2020). Conjugation of Aspergillus flavipes Taxol with porphyrin increases the anticancer activity of Taxol and ameliorates its cytotoxic effects. Molecules.

[5] Liu Y, Guo J, Shen K, Wang R, Chen C, Liao Z, Zhou J (2020). Paclitaxel suppresses hepatocellular carcinoma tumorigenesis through regulating Circ-BIRC6/miR-877–5p/YWHAZ axis. OncoTargets Ther.

[6] Rowinsky EK, Cazenave LA, Donehower RC (1990). Taxol: a novel investigational antimicrotubule agent. JNCI: J Natl Cancer Inst.

[7] Schiff PB, Fant J, Horwitz SB (1979). Promotion of microtubule assembly in vitro by Taxol. Nature.

[8] Straubinger RM, Sharma A, Murray M, Mayhew E (1993). Novel Taxol formulations: Taxol-containing liposomes. J Natl Cancer Inst Monogr.

[9] Malik S, Cusidó RM, Mirjalili MH, Moyano E, Palazón J, Bonfill M (2011). Production of the anticancer drug Taxol in Taxus baccata suspension cultures: a review. Process Biochem.

[10] Kim SY, Lee YM (2001). Taxol-loaded block copolymer nanospheres composed of methoxy poly (ethylene glycol) and poly (ε-caprolactone) as novel anticancer drug carriers. Biomaterials.

[11] Jagtap PG, Baloglu E, Barron DM, Bane S, Kingston DG (2002). Design and synthesis of a combinatorial chemistry library of 7-acyl, 10-acyl, and 7, 10-diacyl analogues of paclitaxel (Taxol) using solid phase synthesis. J Nat Prod.

[12] Exposito O, Bonfill M, Onrubia M, Jane A, Moyano E, Cusidó RM, Palazon J, Piñol MT (2009). Effect of Taxol feeding on Taxol and related taxane production in Taxus baccata suspension cultures. New Biotechnol.

[13] Strobel G, Daisy B (2003). Bioprospecting for microbial endophytes and their natural products. Microbiol Mol Biol Rev.

[14] Stierle A, Strobel G, Stierle D (1993). Taxol and taxane production by Taxomyces andreanae, an endophytic fungus of Pacific yew. Science.

[15] Zhang P, Zhou P-P, Yu L-J. An endophytic Taxol-producing Fungus From Taxus x Media Aspergillus candidus MD3. 2009;293(2):155–9. 10.1111/j.1574-6968.2009.01481.x 19239498

[16] El-Sayed AS, Ali DM, Yassin MA, Zayed RA, Ali GS (2019). Sterol inhibitor “Fluconazole” enhance the Taxol yield and molecular expression of its encoding genes cluster from Aspergillus flavipes. Process Biochem.

[17] El-Sayed AS, El Sayed MT, Nada HS, Hassan AE, Yousef EK (2019). Production and characterization of Taxol as anticancer agent from Aspergillus terreus. J Pure Appl Microbiol.

[18] El-Sayed AS, Abdel-Ghany SE, Ali GS (2017). Genome editing approaches: manipulating of lovastatin and Taxol synthesis of filamentous fungi by CRISPR/Cas9 system. Appl Microbiol Biotechnol.

[19] El-Sayed AS, Hassan MN, Nada HM (2015). Purification, immobilization, and biochemical characterization of l-arginine deiminase from thermophilic Aspergillus fumigatus KJ 434941: anticancer activity in vitro. Biotechnol Prog.

[20] El-Sayed AS, Safan S, Mohamed NZ, Shaban L, Ali GS, Sitohy MZ (2018). Induction of Taxol biosynthesis by Aspergillus terreus, endophyte of Podocarpus gracilior Pilger, upon intimate interaction with the plant endogenous microbes. Process Biochem.

[21] El-Sayed AS, Mohamed NZ, Safan S, Yassin MA, Shaban L, Shindia AA, Shad Ali G, Sitohy MZ (2019). Restoring the Taxol biosynthetic machinery of Aspergillus terreus by Podocarpus gracilior Pilger microbiome, with retrieving the ribosome biogenesis proteins of WD40 superfamily. Sci Rep.

[22] Kusari S, Hertweck C, Spiteller M (2012). Chemical ecology of endophytic fungi: origins of secondary metabolites. Chem Biol.

[23] Staniek A, Woerdenbag HJ, Kayser O (2009). Taxomyces andreanae: a presumed paclitaxel producer demystified?. Planta Med.

[24] Deepika V, Murali T, Satyamoorthy K (2016). Modulation of genetic clusters for synthesis of bioactive molecules in fungal endophytes: a review. Microbiol Res.

[25] Brakhage AA, Schuemann J, Bergmann S, Scherlach K, Schroeckh V, Hertweck C (2008). Activation of fungal silent gene clusters: a new avenue to drug discovery. Nat Compd Drugs.

[26] Bok JW, Hoffmeister D, Maggio-Hall LA, Murillo R, Glasner JD, Keller NP (2006). Genomic mining for Aspergillus natural products. Chem Biol.

[27] El-Dawy EGAEM, Gherbawy YA, Hussein MA (2021). Morphological, molecular characterization, plant pathogenicity and biocontrol of Cladosporium complex groups associated with faba beans. Sci Rep.

[28] El Sayed MT, El-Sayed ASA. Tolerance and mycoremediation of silver ions by Fusarium solani. Heliyon. 2020;6(5):e03866. 10.1016/j.heliyon.2020.e03866 PMC7225397 32426534

[29] Stevenson DW (1992). A formal classification of the extant cycads. Brittonia.

[30] Calonje M, Stevenson DW, Stanberg L: The world list of cycads. In: *10th International Conference on Cycad Biology*, vol. 5. pp. 77–119. Medellín, Colombia 2015: 77–119.

[31] Osborne R, Calonje MA, Hill KD, Stanberg L, Stevenson DW (2012). The world list of cycads. Mem N Y Bot Gard.

[32] Treutlein J, Vorster P, Wink M (2005). Molecular relationships in Encephalartos (Zamiaceae, Cycadales) based on nucleotide sequences of nuclear ITS 1&2, rbcL, and genomic ISSR fingerprinting. Plant Biol.

[33] Negm WA, El-Aasr M, Attia G, Alqahtani MJ, Yassien RI, Abo Kamer A, Elekhnawy E (2022). Promising antifungal activity of Encephalartos laurentianus de wild against Candida albicans clinical isolates: in vitro and in vivo effects on renal cortex of adult albino rats. J Fungi.

[34] Temraz A (2016). Novel illudalane sesquiterpenes from Encephalartos villosus Lehm. Antimicrobial activity. Nat Prod Res.

[35] Eldeghidy A, Abdel-Fattah G, El-Sayed AS, Abdel-Fattah GG (2023). Production, bioprocessing and antiproliferative activity of camptothecin from Aspergillus terreus, endophyte of Cinnamomum camphora: restoring their biosynthesis by indigenous microbiome of C. camphora. Microbial Cell Factor.

[36] El-Sayed AS, Hassan WH, Sweilam SH, Alqarni MHS, El Sayed ZI, Abdel-Aal MM, Abdelsalam E, Abdelaziz S (2022). Production, bioprocessing and anti-proliferative activity of camptothecin from Penicillium chrysogenum, “an endozoic of marine sponge, Cliona sp.”, as a metabolically stable camptothecin producing isolate. Molecules.

[37] Chen A, Frisvad JC, Sun B, Varga J, Kocsubé S, Dijksterhuis J, Kim D, Hong S-B, Houbraken J, Samson RA (2016). Aspergillus section Nidulantes (formerly Emericella): Polyphasic taxonomy, chemistry and biology. Stud Mycol.

[38] Frisvad JC, Samson RA (2004). Polyphasic taxonomy of Penicillium subgenus Penicillium. A guide to identification of food and air-borne terverticillate Penicillia and their mycotoxins. Stud Mycol.

[39] Raper K, FenneIl DI (1965). The Aspergillus fumigatus group.

[40] McPartland TJ, Patil RA, Malone MF, Roberts SC (2012). Liquid–liquid extraction for recovery of paclitaxel from plant cell culture: solvent evaluation and use of extractants for partitioning and selectivity. Biotechnol Prog.

[41] Das A, Rahman MI, Ferdous AS, Amin A, Rahman MM, Nahar N, Uddin MA, Islam MR, Khan H (2017). An endophytic Basidiomycete, Grammothele lineata, isolated from Corchorus olitorius, produces paclitaxel that shows cytotoxicity. PLoS ONE.

[42] Li J-y, Strobel G, Sidhu R, Hess W, Ford EJ (1996). Endophytic Taxol-producing fungi from bald cypress, Taxodium distichum. Microbiology.

[43] Cardellina JH (1991). HPLC separation of Taxol and cephalomannine. J Liq Chromatogr.

[44] Abdel-Fatah SS, El-Batal AI, El-Sherbiny GM, Khalaf MA, El-Sayed AS (2021). Production, bioprocess optimization and γ-irradiation of Penicillium polonicum, as a new Taxol producing endophyte from Ginko biloba. Biotechnol Rep.

[45] Patel JS, Vitoreli A, Palmateer AJ, El-Sayed A, Norman DJ, Goss EM, Brennan MS, Ali GS (2016). Characterization of Phytophthora spp. isolated from ornamental plants in Florida. Plant Dis.

[46] El-Sayed A, Khalaf S, Abdel-Hamid G, El-Batrik M (2015). Screening, morphological and molecular characterization of fungi producing cystathionine γ-lyase. AcEl-ta Biologica Hungarica.

[47] Schoch CL, Seifert KA, Huhndorf S, Robert V, Spouge JL, Levesque CA, Chen W, List FBCA, Bolchacova E, Consortium FB, List FBCA (2012). Nuclear ribosomal internal transcribed spacer (ITS) region as a universal DNA barcode marker for Fungi. Proc Natl Acad Sci.

[48] Kumar S, Stecher G, Tamura K (2016). MEGA7: molecular evolutionary genetics analysis version 7.0 for bigger datasets. Mol Biol Evol.

[49] Barltrop JA, Owen TC, Cory AH, Cory JG (1991). 5-(3-carboxymethoxyphenyl)-2-(4, 5-dimethylthiazolyl)-3-(4-sulfophenyl) tetrazolium, inner salt (MTS) and related analogs of 3-(4, 5-dimethylthiazolyl)-2, 5-diphenyltetrazolium bromide (MTT) reducing to purple water-soluble formazans as cell-viability indicators. Bioorg Med Chem Lett.

[50] Mohamed NZ, Shaaban L, Safan S, El-Sayed AS (2023). Phytochemical and metabolic profiling of the different Podocarpus species in Egypt: potential antimicrobial and antiproliferative activities. Heliyon.

[51] El-Sayed AS, Ibrahim H, Sitohy MZ. Co-immobilization of PEGylated Aspergillus flavipes L-methioninase with glutamate dehydrogenase: a novel catalytically stable anticancer consortium. Enzyme Microb Technol. 2014;54:59–69. 10.1016/j.enzmictec.2013.10.004 24267569

[52] Vermes I, Haanen C, Steffens-Nakken H, Reutellingsperger C (1995). A novel assay for apoptosis flow cytometric detection of phosphatidylserine expression on early apoptotic cells using fluorescein labelled annexin V. J Immunol Methods.

[53] Edgar RC (2004). MUSCLE: a multiple sequence alignment method with reduced time and space complexity. BMC Bioinformatics.

[54] Tamura K, Peterson D, Peterson N, Stecher G, Nei M, Kumar S (2011). MEGA5: molecular evolutionary genetics analysis using maximum likelihood, evolutionary distance, and maximum parsimony methods. Mol Biol Evol.

[55] Samson RA, Noonim P, Meijer M, Houbraken J, Frisvad JC, Varga J (2007). Diagnostic tools to identify black aspergilli. Stud Mycol.

[56] Croteau R, Ketchum RE, Long RM, Kaspera R, Wildung MR (2006). Taxol biosynthesis and molecular genetics. Phytochem Rev.

[57] Volk KJ, Hill SE, Kerns EH, Lee MS (1997). Profiling degradants of paclitaxel using liquid chromatography–mass spectrometry and liquid chromatography–tandem mass spectrometry substructural techniques. J Chromatogr B Biomed Sci Appl.

[58] Kusari S, Singh S, Jayabaskaran C (2014). Rethinking production of Taxol®(paclitaxel) using endophyte biotechnology. Trends Biotechnol.

[59] Subban K, Kempken F (2023). Insights into Taxol® biosynthesis by endophytic fungi. Appl Microbiol Biotechnol.

[60] Zhang P, Zhou P-P, Yu L-J (2009). An endophytic Taxol-producing fungus from Taxus media, Cladosporium cladosporioides MD2. Curr Microbiol.

[61] Deng BW, Liu KH, Chen WQ, Ding XW, Xie XC (2009). Fusarium solani, Tax-3, a new endophytic Taxol-producing fungus from Taxus chinensis. World J Microbiol Biotechnol.

[62] Zhao K, Ping W, Li Q, Hao S, Zhao L, Gao T, Zhou D (2009). Aspergillus niger var. taxi, a new species variant of Taxol-producing fungus isolated from Taxus cuspidata in China. J Appl Microbiol.

[63] Zhang P, Zhou P-P, Yu L-J (2009). An endophytic Taxol-producing fungus from Taxus x media, Aspergillus candidus MD3. FEMS Microbiol Lett.

[64] Xiong Z-Q, Yang Y-Y, Zhao N, Wang Y (2013). Diversity of endophytic fungi and screening of fungal paclitaxel producer from Anglojap yew Taxus x media. BMC Microbiol.

[65] Pandy R, Kumar SS, Suresh P, Annaraj J, Pandi M, Vellasamy S, Sagadevan S (2023). Screening and characterization of fungal Taxol-producing endophytic fungi for evaluation of antimicrobial and anticancer activities. Open Chem.

[66] El-Ghareeb DK, Osman GH, El Baz AF. Isolation, cloning, and overexpression of vip3Aa gene isolated from a local Bacillus thuringiensis. Biocontrol Sci Technol. 2012;22(1):11–21.

[67] Kalimuthu AK, Pavadai P, Panneerselvam T, Babkiewicz E, Pijanowska J, Mrówka P, Rajagopal G, Deepak V, Sundar K, Maszczyk P (2022). Cytotoxic potential of bioactive compounds from Aspergillus flavus, an endophytic fungus isolated from Cynodon dactylon, against breast cancer: experimental and computational approach. Molecules.

[68] Hedayati M, Pasqualotto A, Warn P, Bowyer P, Denning D (2007). Aspergillus flavus: human pathogen, allergen and mycotoxin producer. Microbiology.

[69] Badr H, El-Baz A, Mohamed I, Shetaia Y, El-Sayed ASA, Sorour N. Bioprocess optimization of glutathione production by Saccharomyces boulardii: biochemical characterization of glutathione peroxidase. Arch Microbiol. 2021;203(10):6183–6196. 10.1007/s00203-021-02584-0 34580743

[70] El Baz AF, Shetaia YM, Elkhouli RR. Kinetic behavior of Candida tropicalis during xylitol production using semi-synthetic and hydrolysate based media. Afr J Biotechnol. 2011;10(73):16617–25.

[71] Pandi M, Kumaran RS, Choi Y-K, Kim HJ, Muthumary J (2011). Isolation and detection of Taxol, an anticancer drug produced from Lasiodiplodia theobromae, an endophytic fungus of the medicinal plant Morinda citrifolia. Afr J Biotech.

[72] Chakravarthi BV, Sujay R, Kuriakose GC, Karande AA, Jayabaskaran C (2013). Inhibition of cancer cell proliferation and apoptosis-inducing activity of fungal Taxol and its precursor baccatin III purified from endophytic Fusarium solani. Cancer Cell Int.

[73] Kumaran RS, Choi Y-K, Lee S, Jeon HJ, Jung H, Kim HJ (2012). Isolation of Taxol, an anticancer drug produced by the endophytic fungus Phoma betae. Afr J Biotechnol.

[74] Rajendran L, Rajagopal K, Subbarayan K, Ulagappan K, Sampath A, Karthik G (2013). Efficiency of fungal Taxol on human liver carcinoma cell lines. Am J Res Commun.

[75] Samadi N, Gaetano C, Goping I, Brindley D (2009). Autotaxin protects MCF-7 breast cancer and MDA-MB-435 melanoma cells against Taxol-induced apoptosis. Oncogene.

[76] Woods CM, Zhu J, McQueney PA, Bollag D, Lazarides E (1995). Taxol-induced mitotic block triggers rapid onset of a p53-independent apoptotic pathway. Mol Med.

[77] Chakravarthi B, Das P, Surendranath K, Karande AA, Jayabaskaran C (2008). Production of paclitaxel by Fusarium solani isolated from Taxus celebica. J Biosci.

[78] Bladt TT, Frisvad JC, Knudsen PB, Larsen TO (2013). Anticancer and antifungal compounds from Aspergillus Penicillium and other filamentous fungi. Molecules.

[79] Kamath K, Smiyun G, Wilson L, Jordan MA (2014). Mechanisms of inhibition of endothelial cell migration by taxanes. Cytoskeleton.

[80] Axel DI, Kunert W, Göggelmann C, Oberhoff M, Herdeg C, Küttner A, Wild DH, Brehm BR, Riessen R, Köveker G (1997). Paclitaxel inhibits arterial smooth muscle cell proliferation and migration in vitro and in vivo using local drug delivery. Circulation.

[81] Zhao K, Ping W, Li Q, Hao S, Zhao L, Gao T, Zhou D (2009). Aspergillus niger var. taxi, anew species variant of Taxol-producing fungus isolated from Taxus cuspidata in China. J Appl Microbiol.

[82] El-Sayed AS, Yassin MA, Ibrahim H (2015). Coimmobilization of l-methioninase and glutamate dehydrogenase: novel approach for l-homoalanine synthesis. Biotechnol Appl Biochem.

[83] El-Sayed AS, Shindia AA, Ali GS, Yassin MA, Hussein H, Awad SA, Ammar HA (2021). Production and bioprocess optimization of antitumor Epothilone B analogue from Aspergillus fumigatus, endophyte of Catharanthus roseus, with response surface methodology. Enzyme Microb Technol.

